# Hydrogen-Bonded Colorimetric and Fluorescence Chemosensor for Fluoride Anion With High Selectivity and Sensitivity: A Review

**DOI:** 10.3389/fchem.2021.666450

**Published:** 2021-08-19

**Authors:** Zhifeng Deng, Cheng Wang, Haichang Zhang, Taotao Ai, Kaichang Kou

**Affiliations:** ^1^ School of Chemistry and Chemical Engineering, Northwestern Polytechnical University (NWPU), Xi’an, China; ^2^ National and Local Joint Engineering Laboratory for Slag Comprehensive Utilization and Environmental Technology, School of Materials Science and Engineering, Shaanxi University of Technology (SNUT), Hanzhong, China; ^3^ Key Laboratory of Rubber–Plastic of Ministry of Education (QUST), School of Polymer Science and Engieering, Qingdao University of Science and Technology, Qingdao, China

**Keywords:** fluoride anion sensors, high sensitivity and selectivity, naked eye detection, non-pollution, molecular design, extraction, aqueous phase detection, hydrogen-bond

## Abstract

In recent years, the wide application of fluoride materials has grown rapidly, therefore excessive discharge in the surrounding environment, especially in drinking water and organic effluent, has become a potential hazard to humans, and has even resulted in fluorosis disease. The development of a highly effective and convenient method to recognize fluoride anions in surrounding environments seems necessary and urgent. Among which, the development of a colorimetric and fluorescence fluoride chemosensor with obvious color changing allowing for naked-eye detection with high sensitivity and selectivity is more interesting and challenging. In this minireview, current novel colorimetric and fluorescence chemosensors for fluoride anions by hydrogen-bond interaction are introduced, including obvious color changing by naked-eye detection, high sensitivity and selectivity, non-pollution and fluoride extraction ability, aqueous detection, and other additional functions. Finally, the perspective of the fluoride chemosensor design concept and potential evolution trends are pointed out.

## Introduction

Fluoride anion, as the smallest anion, with the highest charge density and a hard Lewis basic nature, is a significant and essential element for the health of the human body and the development of human society (Xiong et al., 2013; Wu et al., 2019; Wu et al., 2020). Recognition of the importance and side-effect of fluoride anions in biological, medicine, and environmental fields has grown rapidly in recent years. Such as, (i) fluoride, being the basic component for human growth, is easily absorbed by the body while it is excreted slowly (Zhou et al., 2014). As a result, excess fluoride in the human body can lead to bone and thyroid activity disorders (Wade et al., 2010); (ii) fluoride anion also plays a key role in the chemical industry (Xuan et al., 2013), organic synthesis, biological and medical processes (Kleerekoper, 1998; Cametti and Rissanen, 2009), military applications, etc. Therefore, the utilization and emission of fluoride are increasing year by year; (iii) in recent years, environmental pollution by fluoride anions has become one of the key issues which needs be addressed, including the treatment of drinking water (Li et al., 2018). An appropriate amount of fluoride anions existing in the environment is healthy for humans, but excessive fluoride levels in drinking water have been linked to the debilitating bone disease fluorosis, especially in some underdeveloped countries (Kaur and Choi, 2015). Consequently, a highly effective and convenient method to monitor fluoride anions in surrounding environments seems necessary and urgent. The development of highly sensitive and selective fluoride anion sensors with qualitative and quantitative detection appear to be particularly crucial. In the past few years, an abundance of fluoride anion sensors have been designed and obtained based on the theories of the interactions between fluoride anions and Lewis acids, hydrogen bonding, mesoporous silica or silica particles, and so on. Simultaneously, the requirement of fluoride detecting and sensing techniques should be easily carried out, including the electrode method, F NMR analysis, colorimetric and fluorescence sensing, and the electrochemical system (Zhou et al., 2014). Among which, a colorimetric and fluorescence fluoride chemosensor with obvious color changing allowing naked-eye detection with high sensitivity and selectivity is more interesting and promising. Meanwhile, its simple and feasible operation, as well as its function without auxiliary equipment push the colorimetric and fluorescence fluoride chemosensor into the forefront, attracting considerable attention. In this minireview, recently reported colorimetric and fluorescence chemosensors for fluoride anions by hydrogen-bond interaction are introduced. The multi-functional properties of chemosensors subdivided into five aspects, including obvious color changing by naked-eye detection, high sensitivity and selectivity, non-pollution and fluoride extraction ability, aqueous detection, and other functions, are classified and recommended. Finally, the perspective of potential evolution trends are pointed out. We hope that this work will promote new insights and further research studies to boost the development of fluoride anion sensors for next-generation applications.

## Obvious Color Changing by Naked-Eye Detection

A large number of optical chemosensors have been developed for fluoride detection in recent years. Among which, the most common methods used for fluoride detection depend on strong interactions, such as hydrogen bonding, taking place between the sensor and fluoride anions. Recent studies on reported fluoride chemosensors revealed the fact that fluoride anion is an electronegative atom, which could easily react with amino groups (NH units) in the formation of a strong hydrogen bond, meanwhile the deprotonation of the amino moiety (NH) by fluoride anions (inter-molecular proton transfer, IPT) happens (Sun et al., 2017). In most cases, once IPT occurs, the optical spectrum of the sensors will be changed, which could be used for fluoride anion detection by the naked eye and a spectrophotometer. Currently, most reported fluoride anion sensors are realized by the one-photon excitation fluorescence emission spectrum or intensity changes to recognize the fluoride anions.

**FIGURE 1:**
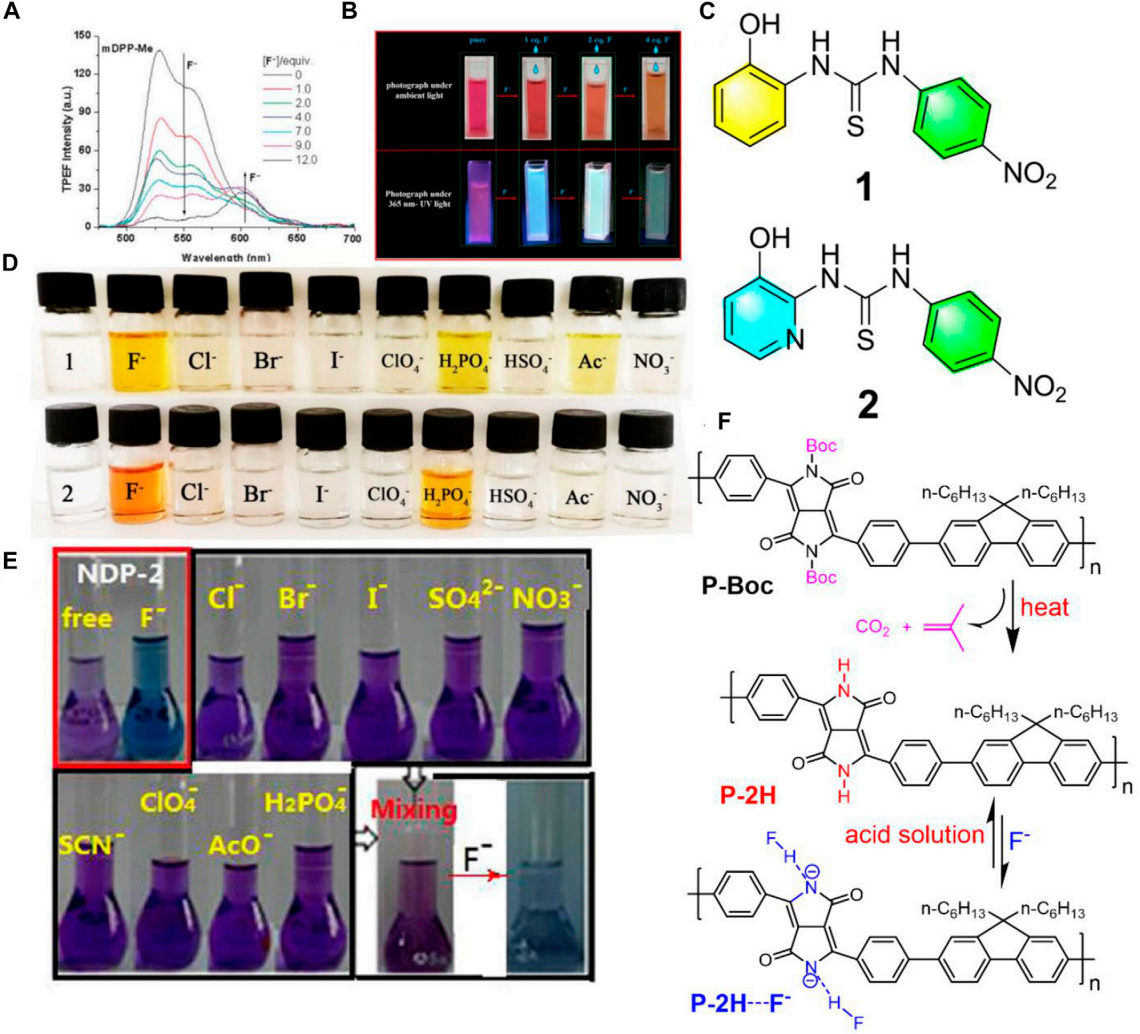
**(A)** Two-photon excitation fluorescence spectra of mDPP in THF (1.0 × 10^−4^ M) with different equivalents of fluoride ions excited at 720 nm under an input laser power of 150 mW (Yang et al., 2013); **(B)** photographs of IDTI solutions with or without additional different eq. Fluoride anions under ambient light or 365 nm UV light (Yuan et al., 2020); **(C)** chemical structures of the fluoride chromogenic sensors of 1 and 2; **(D)** color changes of the sensors in DMSO (2.0 × 10^−4^ mol/L) by the addition of 50 equiv. of different anions (1.0 × 10^−2^ mol/L), respectively (Cao et al., 2020); **(E)** photographs of naphthodipyrrolidone in THF solution before and after the addition of 8 equiv. of different anions under ambient light (Sun et al., 2017); **(F)** chemical structures of P-Boc and P-2H, and the schematic diagram of P-2H sensing fluoride anions (Zhang et al., 2018).

Aside from one-photon excited fluorescence fluoride chemosensors, in 2013, Yang et al. synthesized three *N*-monoalkylated DPP derivatives with mono lactam hydrogen units which were used for two-photon fluorescence chemosensors selectively for fluoride anions (Yang et al., 2013). Compared to the one-photon excited fluorescence fluoride chemosensors, the two-photon fluorescence ones are more interesting, particularly in bio-imaging, which relies on long wavelength light with low energy excitation to obtain signals of short wavelength light change with high energy. These studies showed that a notable new emission band was observed under low fluoride ion concentrations by two-photon excitation (Figure 1A). For the purpose of verifying the nature of the nonlinear absorption of negatively charged species (Ar-DPP-N^-^-Ar), the two-photon emission fluorescence spectra of mDPP-Cl with 6 equiv. of TBAF (Tetrabutylammonium fluoride) were recorded under a constant excitation wavelength (720 nm) with different input laser powers (80–200 mW). The gradually increased output of fluorescence signal intensity with the input of laser power afforded a power-law dependence of exponent 1.96. This study indicated a higher detection sensitivity of the phenyl-DPP to fluoride ions upon two-photon excitation.

However, auxiliary equipment is usually required for fluorescence fluoride chemosensors in the detection of fluoride anions, which can be inconvenient in some urgent cases. In contrast, colorimetric fluoride chemosensors are more favorable, because they can directly monitor fluoride anions by the naked eye. Recently, Yuan et al. developed (3Z,3′Z)-3,3’-(4,4,9,9-tetrakis (4-hexylphenyl)-4,9-dihydro-s-indaceno (1,2-b:5,6-b’)dithiophene]-2,7-diylbis (methan-1-yl-1-ylidene)) bis(indolin-2-one) (IDTI) and used it as a fluoride chemsensor (Yuan et al., 2020). Once the IDTI detected fluoride in the THF solution, the color changed directly from purple to green under UV light, as well as from red to yellow under ambient light which were both easy to observe with the naked eye (Figure 1B). Compared to Yang’s work (Yang et al., 2013), the IDTI fluoride chemsensors seem more favorable due to their dispensable additional equipment and noticeable color variations. Normally, the detection limitation of colorimetric fluoride chemsensors is around 10^−5^ M. Interestingly, the dyes of IDTI could quantitatively analyze fluoride concentrations with a detection limit of as low as 1 × 10^−7^ M.

## High Sensitivity and Selectivity

Instead of introducing single functional amino units (NH), bringing in two or more functional groups into the molecular main chain to detect fluoride anions is also more important and effective. Cao et al. synthesized two new chromogenic sensors 1-(2-hydroxyphenyl)-3-(4-nitrophenyl) thiourea (1) and 1-(3-hydroxypyridin-2- yl)-3-(4-nitrophenyl) thiourea (2) bearing nitrophenyl and thiourea groups (Cao et al., 2020) (Figure 1C). Based on the existence of amino and hydroxyl moieties as receptors, obvious color changes from colorless to yellow in solution containing fluoride anions was apparent (Figure 1D). These two sensors were quite sensitive to fluoride anions, which showed a detection limit of 5.45 × 10^−7^ M. This highly sensitive property could be ascribed by the fact that these sensors contained two functional groups of amino and OH possessing protons, which could bind the fluoride anions and then be deprotonated by the fluoride. Although these two sensors showed high sensitivity to fluoride anions, they exhibited poor selectivity to fluoride anions as the color only changed once when met with H_2_PO_4_^−^ or Ac^−^ (Figure 1D).

Good fluoride chemosensors can not only have high sensitivity for fluoride anions, but also need to have high selectivity. Zhang et al. introduced naphthodipyrrolidone (NDP) derivatives with two lactam units as fluoride anion receptors, which afforded no noticeable color change upon adding 8 equiv. of single or the mixture of various anions, such as Cl^−^, Br^−^, I^−^, SO_4_^2−^, NO_3_^−^, SCN^−^, ClO_4_^−^, AcO_4_^−^, and H_2_PO_4_^−^, while a rapid and noticeable color change from purple to blue was observed after adding fluoride anions into the mixture solutions (Sun et al., 2017) (Figure 1E). This indicated that the NDP is a highly selective fluoride anion sensor with no interference from other anions. Other highly sensitive and selective fluoride chemosensors based on diketopyrrolopyrrole (Qu et al., 2012; Zhang et al., 2013), loutonin (Mohanasundaram et al., 2020), carbon dots (Liu et al., 2021), and so on have been manufactured.

## Non-pollution and Fluoride Anion Extraction Capacity

Most of the representative organic fluoride chemosensors were soluble in solvents, which meant that these chemosensors could only be used once or were unrecyclable. In addition, some of the chemosensors could cause secondary pollution. Hence, it is desirable and challenging to develop fluoride anions sensors with a high performance of nonpolluting capacity, repeatability, and reversibility. In 2018, Feng et al. designed a monomer with N-monoalkylated DPP as the core, and substituted carbazole groups as molecule ends (Feng et al., 2018). Upon electrochemical polymerization in the monomer solution, the cross-linking polymer film was deposited on the surface of ITO glass. This film revealed insolubility in most common organic solvents, which prompted its environmentally friendly performance and did not cause secondary pollution. This study verified that the polymer film could detect fluoride anions in organic solvents with high sensitivity (the detection limit was 4.1 × 10^−8^ M), as well as repeatability. Films synthesized by electrochemical polymerization, which are mostly of a small area, would limit its development and application in a large area.

A high amount of fluoride in the environment is hazardous and even toxic. Therefore, a fluoride chemosensor possessing a fluoride anion extraction capacity without pollution is valued by the scientific and industrial society. Recently, Zhang et al. synthesized a soluble alternating copolymer (P-Boc) consisting of fluorene and N-butoxycarbonyl substituted-DPP (Zhang et al., 2018). The new polymer film (P-2H) with multiple potential lactam units could be obtained and appeared upon heating the spin-coated P-Boc film through the removal of the t-butoxycarbonyl groups (Figure 1F). The P-2H film was insoluble and exhibited a remarkable naked-eye fluoride detection by a visible color change from red to green in common organic solution. Interestingly, the fluoride anions were bonded by the P-2H film (Figure 1F) that could be easily removed by immersing in acidic solutions, therefore P-2H film can be used as a reusable fluoride sensor. This indicated that the P-2H film was not only a renewable fluoride anion chemosensor, but also a fluoride anion extractor. To the best of our knowledge, this is the first reported fluoride chemsensor with renewable ability and efficiency, which is valuable due to its non-pollution aspect, especially fitting to the current trend of green chemistry.

## Aqueous Phase Detection

Fluoride anions play the key role in an environmentally friendly society, animal activities, and even in human health. A moderate fluoride anion intake is beneficial and prevents dental caries and rehabilitation osteoporosis (Udhayakumari, 2020). However, an excess amount of fluoride may cause serious skeletal fluorosis (Zhang et al., 2017) and induce kidney and liver damage (Shao et al., 2019). Currently, fluoride anions as psychotropic substances and some pesticides are being increasingly used (Wu et al., 2017). Consequently, it has become a normal pollutant even in soil and our daily drinking water. Up to now, traditional colorimetric and fluorescence chemosensors are mainly used in the organic phase, which display no reaction with fluoride anions in aqueous media and living cells. Hence the development of high-performance fluoride anions sensors in the aqueous phase is urgently needed and challenging.

**FIGURE 2:**
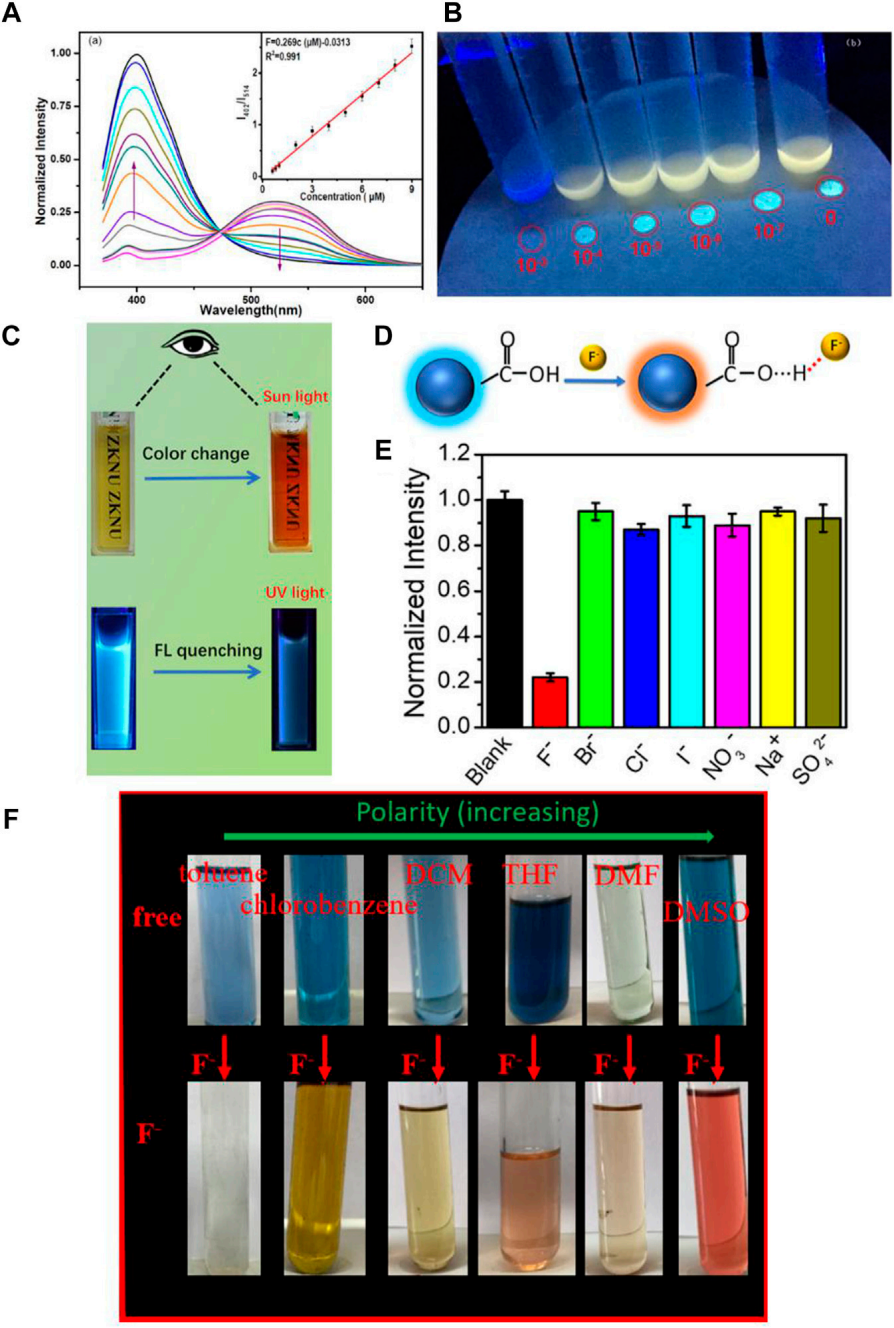
**(A)** Fluorescent emission changes of the sensors (10 μ mol L^−1^) in the presence of increasing amounts of fluoride anions in 90% MeCN-H_2_O (v/v); **(B)** solid-state fluorescence response of the sensors with different concentrations of fluoride under UV illumination at 365 nm (Yuan et al., 2020); **(C)** photograph of fluorescence/colorimetric detection of fluoride anions; **(D)** the proposed structure of CDs and mechanism for identifying fluoride anions; **(E)** normalized fluorescence intensity of the CDs in the presence of various individual ions (Liu et al., 2021); **(F)** the photographs of ABDF in various solutions before and after the addition of 4.5 eq. of fluoride anions (Deng et al., 2020).

In 2020, Tian et al. successfully synthesized a kind of isoquinolinium salt with good fluoride anion detection performance in the aqueous phase (Zhao et al., 2020). In the water phase, fluoride anions could react with isoquinolinium salts which resulted in a significant fluorescent emission spectra change with the emission peak blue shifting from 520 to 400 nm, simultaneously with a color change from yellow to blue (Figures 2A,B). Surprisingly, these kinds of sensors could selectivity and quantitatively recognize fluoride anions with the limitation as low as 3.7 × 10^−9^ M in a 90% MeCN-H_2_O (v/v) phase, which is one of the best reported sensitive fluoride chemosensors. Isoquinolinium salts as a chemosensor can be used to recognize fluoride in our daily drinking water, since the limitation concentration of fluoride anions in drinking water is 211 μM determined by the United States Environmental Protection Agency (EPA). In this work the author further analyzed the fluoride anion detection mechanism by mass spectrometry and nuclear magnetic resonance technique. Fluoride could quickly react with the sensor resulting in the cascade reaction of hydroxyl deprotection and 1,6-oxidation elimination, accompanied with significant fluorescence phenomenon enhancement as well as the presence of fluorescence spectra and the color change.

In 2021, Tian et al. developed a kind of fluorescent carbon dot (CDs) with good water solubility by the green and simple hydro-thermal method from wheat straw without any additives and surface passivation (Liu et al., 2021). The synthesized CDs can be used as fluoride chemsensors in the aqueous phase with color changing from yellow to red under ambient light and emission quenching under UV-light, separately, by adding fluoride anions (Figure 2C). The color change could be ascribed to the hydrogen bond formation between the fluoride anions and the hydroxyl groups of the CDs (Figure 2D). The CDs showed not only high selectivity for the fluoride anions in aqueous solution, but also high sensitivity with a detection limit of about 49 μM (Figure 2E). Furthermore, the studies also indicated that CDs could also be applied in the biological system such as in living cells.

That fluoride chemsensors work in the aqueous phase is crucial, since it has potential in many applications in our daily life such as recognizing the concentration of fluoride anions in the river, in our drinking water, even in living cells. Development of fluoride anion sensors working in aqueous media with high sensitivity and selectivity are urgently necessary and always a challenge.

## Other Additional Functions

During organic synthesis, fluoride anions might appear in organic media such as fluoride anion-containing pesticides and waste organic liquor (Clark, 1980; Feng et al., 2018). In most cases, those organic solvents are different, and some of them are toxic. Fluoride anion sensors could detect fluoride anions as well as types of organic solvents, which are prospective. In 2020, Deng et al. exploited a new aminobenzodifuranone (ABDF) which showed solvatochromic behavior in different organic solvents with various polarities (Deng et al., 2020). Interestingly, ABDF could not only qualitatively and quantitatively detect fluoride anions with high selectivity and sensitively (detect limitation of 5.0 × 10^−7^ M), but could also detect the polarity and type of solvents containing fluoride through various color changes (Figure 2F). This work provided a new design concept for fluoride chemosensors using solvatochromic chemosensors, which could not only detect fluoride anions, but could also additionally detect organic solvents. The same year, X. Wang and coauthors synthesized rare-earth metal-organic frameworks (RE-MOFs) (Wang et al., 2020). RE-MOFs were successfully used for fluoride detection in bottled water and Chinese green tea (Biluochun) with acceptable results. In addition, it was also used to recognize the pH of the aqueous solution. Development of high performance fluoride chemsensors with multi-functions are the current trend.

## Conclusions and Outlook

Fluoride anions are significant and essential for the health of the human body and the development of human society. Too little or an excessive amount of fluoride elemental in the human body is dangerous and unhealthy. Hence, a highly effective and convenient method to monitor fluoride anions in the surrounding environments seems necessary and urgent. Among those reported fluoride chemosensors, the colorimetric and fluorescence fluoride chemosensor with obvious color changing allowing naked-eye detection with high sensitivity and selectivity is more interesting and in line with the current trend. In this minireview, the current novel colorimetric and fluorescence chemosensors for fluoride anions by hydrogen-bond interaction are introduced. We highlighted advances made in the development of organic dye-based fluoride anion sensors from the following facts: i) A sensor with an obvious color change capacity under ambient light or UV irradiation could afford easy and simple fluoride detection without using auxiliary equipment; ii) a sensor with high sensitivity and selectivity could fulfill fluoride detection avoiding external interference, even at low fluoride anion concentrations; iii) a sensor with insolubility could obtain a non-pollution environmentally friendly ability and even with a fluoride anion extraction capacity; iv) sensors with aqueous phase detection ability could be applied to our daily life, particularly in drinking water and living cells, in a great way; and v) fluoride chemsensors with multi-functions, such as the detection of organic solvents and the pH of aqueous solutions, are the requirement from a social development standpoint and on trend.
